# Biomarkers and Mechanisms in the Early Assessment of Childhood Obesity from a Multidisciplinary Perspective—A Narrative Review

**DOI:** 10.3390/medicina61040607

**Published:** 2025-03-27

**Authors:** Dana Elena Mindru, Laura Iulia Bozomitu, Dana Teodora Anton Păduraru, Elena Țarcă, Antoanela Curici, Eva Maria Elkan, Lăcrămioara Ionela Butnariu, Dan Cristian Moraru, Cosmin Diaconescu, Alina Costina Luca

**Affiliations:** 1 Department of Mother and Child Medicine, Faculty of Medicine, University of Medicine and Pharmacy “Gr.T.Popa”, 700115 Iasi, Romania; mindru.dana@umfiasi.ro (D.E.M.); laura.bozomitu@umfiasi.ro (L.I.B.); dana.anton@umfiasi.ro (D.T.A.P.); acluca@yahoo.com (A.C.L.); 2 Emergency Clinical Hospital for Children “Sfanta Maria” Iasi, 700309 Iasi, Romania; 3 Department of Surgery II—Pediatric and Orthopedic Surgery, Faculty of Medicine, University of Medicine and Pharmacy “Gr.T.Popa”, 700115 Iasi, Romania; 4 Department of Cellular and Molecular Biology and Histology, “Carol Davila” University of Medicine and Pharmacy, 050474 Bucharest, Romania; antoanela.curici@umfcd.ro; 5 Department of Morfofunctional Sciences, Faculty of Medicine and Pharmacy, “Dunărea de Jos” University of Galați, 47 Domnească Street, 800008 Galați, Romania; eva.elkan@ugal.ro; 6 Department of Medical Genetics, Faculty of Medicine, University of Medicine and Pharmacy “Gr.T.Popa”, 700115 Iasi, Romania; ionela.butnariu@umfiasi.ro; 7 Department of Plastic Surgery and Reconstructive Microsurgery, “Sf. Spiridon” Emergency County Hospital, 700111 Iasi, Romania; cristian-dan.moraru@umfiasi.ro; 8 Faculty of Medicine, Doctoral School of Medical Sciences, University of Medicine and Pharmacy “Gr.T.Popa”, 700115 Iasi, Romania; diaconescu.cosmin@d.umfiasi.ro

**Keywords:** obesity, adipose tissue, biomarkers, RAS, KKS, microbiota

## Abstract

Obesity has been the subject of research focused on preventive policies among the young population due to epidemiological studies which have shown devastating figures in recent years in terms of the incidence and prevalence of this condition. A number of previously known biomarkers have proven useful in the early diagnosis of complications associated with obesity, while others remain in the study stage. The intestinal microbiota are also relevant in the secondary prevention of obesity complications, another area that has turned into a hot topic of current research. The primary goal of this review is to highlight markers and mechanisms that can enhance specialists’ understanding of obesity assessment and its systemic complications. Salivary markers have been proven useful in the evaluation of obesity, with the advantage of being low-cost and easy to sample. Another interesting topic is the role of the renin–angiotensin and the kallikrein–kinin systems in obesity-related systemic complications. One well-known fact is the connection between obesity and high blood pressure, which is closely related to these systems. This paper also explores the link between gut microbiota and adiposity, particularly the potential of the Firmicutes/Bacteroidetes ratio as a useful biomarker.

## 1. Introduction

Obesity is defined as an excessive accumulation of fat that is detrimental to health and wellness, driven by a positive energy balance resulting from excessive caloric intake and/or inadequate physical activity, and is influenced by various genetic, behavioral, and environmental factors [1]. The prevalence of childhood obesity is increasing worldwide and is strongly associated with metabolic abnormalities, including inflammation, insulin resistance, and dyslipidemia. Obesity in childhood is a public health problem of global proportions, representing a state of chronic low-grade inflammation resulting from visceral fat accumulation, leading to complications such as metabolic syndrome [2].

Obesity is now a major global problem, affecting all age groups. In recent decades, the prevalence of overweight adolescents and children has seen an alarming increase, rising from just 4% in 1975 to over 18% in 2016. According to the World Obesity Federation’s 2019 estimates, it is projected that by 2025, there will be 206 million adolescents and children aged 5–19 years diagnosed with obesity, with the number expected to rise to 254 million by 2030 [3]. All 42 countries considered are expected to have at least 1 million obese children by 2030, with the top five ranked countries being China, India, the US, Indonesia, and Brazil. It is worth noting that only seven of these 42 countries are considered wealthy nations. In many of these countries, the prevalence of severe obesity among children has increased, despite the overall prevalence of obesity remaining stable. According to a survey conducted in European countries, about 25% of children with obesity were classified as severely obese, which has significant consequences for the provision of clinical obesity services, as these children require specialized and intensive treatment [3]. Data presented in the WHO European Regional Obesity Report 2022 indicate that nearly one in three children are affected by overweight and obesity in the WHO European Region, with 29% of boys and 27% of girls impacted [4].

In Romania, the situation of pediatric obesity is as worrying as in the rest of the world. In 2018, the prevalence of childhood obesity was 18.5%, marking an alarming increase from 2010, when it was around 10%. The forecasts for 2030 are also pessimistic: the WHO estimates that 500,000 children aged between 5 and 18 in Romania will be affected by obesity [5]. In addition, in our country, many parents and grandparents do not recognize that overweight or obesity is a problem, which is why they are not interested in implementing weight loss measures, indicating a lack of medical education as a potential underlying issue [6,7].

Biomarkers are biological indicators of various diseases in the human body, with certain categories of biomarkers being associated with obesity. Among these, the most commonly and widely studied are serum, salivary, and fecal biomarkers.

The purpose of this article is to present markers and mechanisms that can enhance specialists’ understanding of obesity assessment and its systemic complications.

## 2. Biomarkers Currently Related to Obesity

In the table below, the biomarkers associated with obesity through different mechanisms are presented (Table 1).

In recent decades, the role of serum biomarkers in relation to obesity has been thoroughly studied, and it has been concluded that C-reactive protein, ferritin, white blood cell count, and red cell distribution width are direct inflammatory biomarkers of obesity-related inflammation in children [8].

Adipose tissue synthesizes complementary components and generates activation products in direct proportion to its volume. This supports the notion that the complement system plays a pivotal role in triggering the low-grade inflammation commonly associated with obesity. The link between adiposity and immune activation underscores the systemic nature of inflammation in obesity. Research indicates that this phenomenon is observable even in children and adolescents who appear healthy but have excess adiposity. Adipose tissue is also a source of C-reactive protein (CRP), a marker used to assess both inflammation and cardiovascular risk. CRP has been found to increase significantly with excess adipose tissue and may play a role in regulating CRP levels through IL-6 production [9].

In the early 21st century, elevated secretion of cytokines, particularly TNF-α and IL-6, which play a role in insulin resistance, was shown to be associated with the infiltration and accumulation of macrophages in adipose tissue. The central role of the macrophage as an immune cell contributing to adipose tissue inflammation has become more relevant in recent years, along with morphological changes in adipocytes and alterations in the quantity and composition of immune cells, leading to the stimulation of a chronic condition. The inflammatory cascade, as a primary immune response, is primarily mediated by macrophages in synergy with the toll-like receptor family, particularly toll-like receptor 4 (TLR4), which binds the lipopolysaccharide (LPS) ligand, initiating the signaling mechanism.

Regarding acute-phase reactants, elevated CRP levels in obese individuals appear to be not only a risk marker but also an indicator of an active role in cardiovascular disease and are associated with myocardial infarction, further highlighting the widespread relationship between inflammation and heart disease [10].

Adipose tissue has been shown to facilitate both the initiation and maintenance of systemic inflammation. Adipose tissue-induced inflammation triggers a complex immune response, beginning with neutrophil activation in the early stages, followed by macrophage recruitment and mast cell polarization. This cascade of immune cell involvement contributes to the progression of metabolic syndrome, highlighting the intricate relationship between immune responses and the development of obesity-related metabolic disorders.

A study of 6700 patients discovered positive correlations between waist circumference and the levels of leukocytes, lymphocytes, neutrophils, platelets, and mean platelet volume. Furthermore, a separate study involving 223 subjects showed that an increase in BMI results in a rise in leukocytes, lymphocytes, platelets, and neutrophils.

The neutrophil-to-lymphocyte ratio (NLR) is a recently identified, cost-effective marker for detecting subclinical inflammation that correlates with C-reactive protein (CRP) levels. This valuable marker has been linked to multiple inflammatory conditions, cardiovascular disease, and cancer. NLR has been shown to be directly associated with the degree of inflammation [11].

The primary distinction between obesity-induced systemic inflammation and the classical inflammatory pathway is the central role of adipose tissue. Adipokines produced by adipose tissue are involved in an autocrine and paracrine manner in the management of energy consumption, insulin sensitivity, glucose and lipid metabolism, endothelial function and inflammation [10].

Adipokine dysregulation has become evident as a feature of chronic inflammation. Adiponectin and leptin are two hormones produced by adipose tissue. Adiponectin inhibits the nuclear factor kappa B (NF-κB) signaling mechanism and also protects against insulin resistance and atherosclerosis. Additionally, it antagonizes TNF-α, and its concentration decreases in obese individuals. It is secreted by adipocytes in inverse proportion to the amount of lipid stored and appears to influence insulin sensitivity. Low adiponectin levels and increased insulin resistance are also known to be associated with the clinical features of metabolic syndrome [12], as obesity and metabolic syndrome are characterized by decreased serum adiponectin levels in parallel with increased concentrations of circulating leptin.

Hypoadiponectinemia and hyperleptinemia are observed in both adults and children with obesity, and the adiponectin/leptin ratio has been suggested as a sensitive marker for metabolic syndrome in children and adolescents [2].

Leptin and adiponectin have been associated with changes in immune function and glucose metabolism [13]. Leptin, acknowledged as an appetite-suppressing hormone that increases during inflammation, is also involved in the Th1 immune response, which sustains the inflammatory state [12]. The identification of leptin is the most significant discovery in obesity research, as it has helped unravel the architecture of neuroendocrine circuits that control appetite and energy homeostasis [14]. Leptin mainly acts in the hypothalamus to control food intake, satiety, and energy consumption. It also has pro-inflammatory effects, and high levels of leptin are associated with the development of insulin resistance and cardiovascular disease. Sleep deprivation has been shown to decrease circulating leptin levels and increase ghrelin levels, as well as self-reported hunger. Ghrelin is the ‘hunger hormone’ that activates the Y-peptide and Agouti gene-related peptide to stimulate appetite. The harmony between adiposity and appetite stimulation is managed by neuroendocrine interaction between the gut, brain, and adipose tissue [15].

Recent studies show that children with obesity are prone to have higher circulating leptin, which drops with decreasing BMI. This is due to insensitivity to circulating leptin and disruption of leptin signaling in the hypothalamus. Low leptin levels, which may be related to low body weight, have been associated with high physical activity. Physical activity is already known to be the most effective non-pharmacological remedy for treating overweight or obesity, as it increases energy expenditure. Sirico et al. demonstrated in their meta-analysis that exercise alone, without accompanying dietary or lifestyle modifications, reduced plasma levels of leptin and IL-6, indicating a decrease in systemic inflammation associated with obesity [16]. Furthermore, Belcher et al. found that leptin levels predicted a decrease in physical activity at the start of puberty, regardless of central adiposity. Their study highlighted that higher leptin levels were associated with lower activity levels in early puberty [17,18].

The octanoyl peptide hormone ghrelin controls appetite and glycemic control. Desacyl-ghrelin eliminates some effects of ghrelin but does not bind to its receptor. Some evidence shows that plasma levels of these peptides differ in adults with obesity, but their levels in childhood obesity remain poorly studied. There are, however, studies that suggest ghrelin tone is increased in childhood obesity due to decreased plasma levels of desacyl-ghrelin and LEAP2, and that desacyl-ghrelin is linked to insulin resistance, particularly in overweight or obese children [10].

Adiponectin is a prominent protein produced by adipose tissue that regulates insulin sensitivity through its action on the liver and muscles. The recent demonstration that adiponectin enters the cerebrospinal fluid to act centrally in regulating body weight extends the role of this hormone beyond its peripheral action on glucose metabolism. It is worth noting that adiponectin appears to cause weight loss through an enhancement in energy expenditure, as food intake is not affected by either central or systemic adiponectin. Therefore, reduced plasma adiponectin levels in obese individuals may serve as a key pathophysiological mechanism [14]. Adiponectin correlates with reduced total body fat mass and encourages insulin sensitivity. Obesity and metabolic syndrome are recognized by decreased serum adiponectin in parallel with intensified circulating leptin concentrations [2]. Higher levels of resistin, an adipokine produced by macrophages and involved in the pathogenesis of inflammation, were also reported in obese and overweight children compared to normal-weight children [19]. Also, adiponectin is a cytokine secreted by adipocytes with anti-inflammatory, antioxidant, and cardioprotective roles. It does not alter dietary intake but causes weight loss by increasing energy expenditure and regulates plasma glucose, lipid, and insulin levels [20].

## 3. Salivary Biomarkers

Saliva is a complex biological fluid produced by the salivary glands, with multiple functions in maintaining a healthy body. In recent years, it has succeeded in helping identify and measure many biomarkers associated with certain pathologies. Salivary samples convey a clearer picture at the cellular level by identifying biologically active compounds compared to blood samples, which detect compounds bound to protein fractions in the bloodstream, the disadvantage being the low levels of biomarkers, reported in pico- and nanograms, making few tests eligible [21].

Salivary markers are viable options for early diagnosis in several pathologies, including childhood obesity. Their use is increasing in Romania due to their low cost and good applicability, thus becoming a satisfactory alternative when blood collection is difficult, especially in pediatric patients [22]. There are some disadvantages that may influence the decision to opt for this method of investigation, including lower salivary concentrations than in serum, easy contamination of saliva with different compounds, low detection limits, interference with food and drink ingested before collection, and difficult cooperation with young pediatric patients who may not understand the instructions [22,23].

Hend Alqaderi et al. conducted a study on a cohort of 353 adolescents followed for 7 years. They were assessed in 2012, 2014, and 2019 by measuring BMI, collecting salivary markers, serum markers, and blood glucose determination. Among the markers followed were the pro-inflammatory cytokines IL-6, IL-8, IL-10, leptin, CRP, insulin, VEGF, MCP-1, and adiponectin (measured in saliva and serum). Results showed that insulin, CRP, and adiponectin play an important role in differentiating patients diagnosed with obesity and intermediate hyperglycemia. For each increase in CRP and insulin levels by one unit, patients showed an increased BMI value of 3.2–3.5 kg/m^2^, with adiponectin decreasing. Hyperinsulinemia is a decisive factor in the development of insulin resistance, which underlies the onset of childhood obesity as well as the development of type 1 diabetes. Salivary insulin levels correlated with serum insulin levels, although salivary levels were up to 10 times lower, thus being a predictor of hyperglycemic status [24].

Another 2019 study examines salivary IL-6 and leptin levels using stochastic sensors, given their low values in saliva at the limit of detection. The study involved 22 children, 17 of whom were diagnosed with obesity or overweight. Salivary and blood samples were collected to measure cholesterol, blood glucose, HDL, triglycerides, and liver enzymes, while BMI was assessed using percentiles. The results revealed that children with obesity or overweight exhibited a significant increase in salivary IL-6 levels, up to 4.5 times higher than their normal-weight counterparts, with only two overweight children showing normal IL-6 values. Additionally, salivary leptin levels were three times higher in the obese and overweight groups compared to those with normal weight [25].

Another salivary marker (besides adiponectin) possibly involved in the diagnosis of childhood obesity is fetuin-A. Fetuin-A is a glycoprotein produced by hepatocytes as well as endocrine, autocrine, and paracrine glands. It plays a role in appetite control and adipogenesis, influences energy intake, and modulates insulin receptors. It lowers insulin levels and regulates hepatic glucose secretion [26]. A 2022 study investigated the possible involvement of adiponectin and fetuin-A in the early diagnosis of childhood obesity. The study included 76 patients aged 6 to 10 years, classified according to BMI value into normal weight, overweight, and obese. Saliva samples were taken in the morning following a 12 h fasting period. Overweight and obese patients showed increased levels of fetuin-A and insulin, while adiponectin was inversely correlated with BMI [27]. This study supports the hypothesis of increased salivary insulin levels in the high-BMI patient group, as observed in the study by Hend Alqaderi et al. [24]. Determination of fetuin-A and salivary adiponectin is thus becoming a possible option in the early diagnosis of childhood obesity, with more studies needed in the future.

The contribution of uric acid in the development of metabolic syndrome has been intensively studied over time, with elevated uric acid values being correlated with the subsequent development of obesity in the adult population. Uric acid is the final product of the metabolism of nucleic acids, purine bases, and nucleoproteins [28,29]. A 2023 peer review examined 161 articles from 2015–2022 on the importance of salivary markers associated with pediatric pathology. The review was divided into seven sections: pulmonary pathology, gastrointestinal, normal development of the pediatric patient, infectious diseases, neuropsychiatry, dental conditions, and autoimmune diseases. Increased salivary uric acid has been correlated with insulin-resistant status and the development of metabolic syndrome in pediatric patients [28]. The presence of hepatic steatosis in the high-BMI group may be a risk factor associated with childhood obesity, which can be detected by salivary uric acid determination.

## 4. Role of RAS and KKS Systems in Obesity-Related Systemic Complications

Research points to the onset of atherosclerosis taking place during early life stages, and the occurrence of atherogenic dyslipidemia is becoming more common in children and adolescents affected by obesity. It is characterized by hypertriglyceridemia, increased very low-density lipoprotein cholesterol (VLDL-C), and decreased HDL-C, and its association with metabolic syndrome also increases the risk of cardiovascular disease [2].

**Figure 1:**
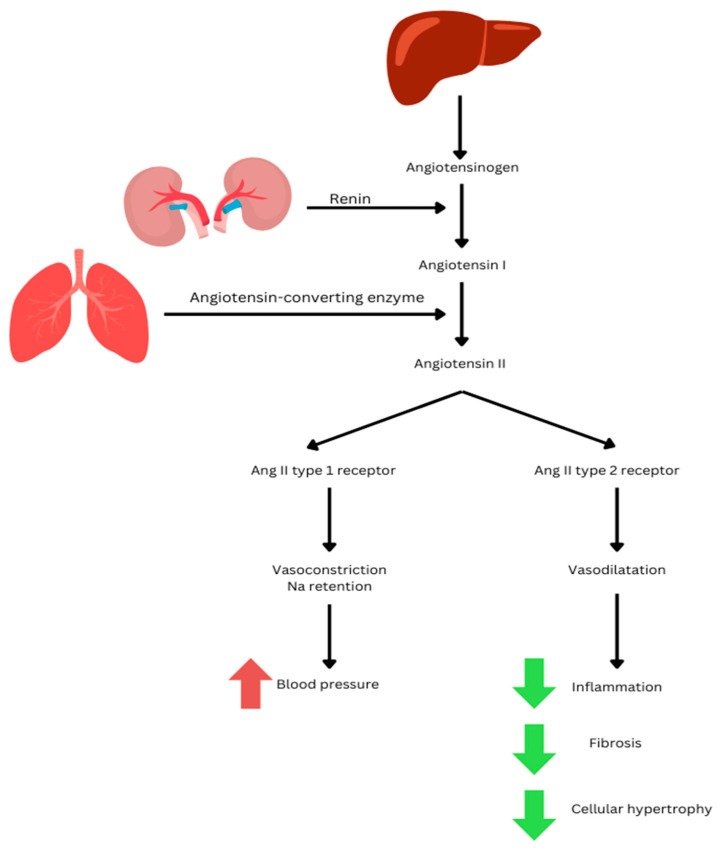
Classical RAS pathway.

Obesity is closely associated with high blood pressure and cardiovascular disease. Various central and peripheral dysfunctions have been recognized that could contribute to the onset or persistence of hypertension in individuals with obesity. Among the key factors are the stimulation of the renin–angiotensin–aldosterone system and the sympathetic nervous system [14]. The renin–angiotensin system (RAS), which plays a crucial role in regulating blood pressure and metabolism, consists of traditional pathways responsible for vasoconstriction, fibrosis, and inflammation, alongside alternative pathways that produce opposite physiological effects, such as vasodilation, anti-fibrotic, and anti-inflammatory actions. It is described as being localized in organs such as the heart, pancreas, brain, and adipose tissue. In the classical RAS pathway, renin cleaves angiotensinogen (AGT) to produce angiotensin I (Ang I), which is subsequently converted into angiotensin II (Ang II) by angiotensin-converting enzyme (ACE). Angiotensin II (Ang II) exerts its effects by interacting with either the Ang II type 1 receptor, which triggers vasoconstriction and sodium retention, thereby promoting an increase in blood pressure, or the Ang II type 2 receptor, which mediates vasodilatation and mitigates processes such as inflammation, fibrosis, and cellular hypertrophy [2] (see Figure 1).

RAS and its main active element, angiotensin II, are crucial in regulating energy homeostasis. The detection of classical RAS components in adipose tissue sparked interest in the interactions between RAS and energy balance. Adipose tissue plays an important role in regulating systemic RAAS, with increasing evidence suggesting that angiotensin II significantly contributes to the heightened inflammatory risks associated with obesity and cardiometabolic disorders [30].

There is evidence that RAS plays a role in the pathophysiology of several diseases, e.g., diabetes, Alzheimer’s, metabolic syndrome, and obesity, in addition to its well-established contribution to the onset and persistence of hypertension. RAS is actually more than just a cascade consisting of a precursor protein (angiotensinogen), two proteases (renin, angiotensin), and an angiotensin II peptide. The discovery of new axes of the RAS system, such as ACE2/Ang1-7/Mas and Ang A/Alamandine/MrgD, which present peptides with similar or opposite functions to angiotensin II, highlights the complex balance of the system, as there is a counter-regulation of the axis to ensure homeostasis of the body. In addition, there is an important interaction between RAS and the kallikrein–kinin system (KKS), which is critical for organ function.

Studies have shown that RAS and KKS are modulated in obesity. There is a positive correlation between low levels of the vasodilator peptides bradykinin (BK) and Ang1-7 and increased body weight. Also, increased concentrations of Ang I and des-Arg9BK, an inflammatory kinin, were found in obese youth [31]. In the KKS system, kallikrein cleaves kininogen to produce bradykinin, which is subsequently converted into des-Arg9-bradykinin by the action of carboxypeptidases M and N (also referred to as kininase II). Bradykinin and des-Arg9-bradykinin are involved in several biological processes, including vasodilation, inflammation, pain, increased vascular permeability, and natriuresis [2].

In a study by Fernandes et al., a thorough analysis of circulating RAS and KKS peptides in healthy adolescents was conducted to identify potential biomarkers of obesity. Clinically healthy adolescent volunteers (ages 11 to 17) were grouped into normal-weight subjects, obesity caused by white adipose tissue, obesity caused by brown adipose tissue, and morbidly obese subjects according to BMI. High-performance liquid chromatography quantified the circulating concentrations of Ang I, Ang II, angiotensinogen, Ang1-7, bradykinin, and des-Arg9-bradykinin. The circulating RAS analysis revealed a positive correlation between Ang I and BMI, while angiotensinogen and Ang1-7 showed a negative correlation with BMI. On the other hand, circulating KKS analysis showed that bradykinin had an inverse relationship and des-Arg9-bradykinin a positive relationship with BMI. Negative angiotensinogen, Ang1-7, and des-Arg9-bradykinin correlations with BMI strongly imply reduced ACE2 activity in obesity. ACE2 is broadly expressed across various organs, including the heart, liver, kidney, pancreas, adipose tissue, skeletal muscle, and intestine, among others. While the specific tissues responsible for the observed decrease in ACE2 activity in obesity remain uncertain, it is plausible to speculate that adipose tissue-derived ACE2 may contribute to these systemic effects. This conjecture is supported by emerging evidence showing a negative correlation between ACE2 mRNA expression in subcutaneous adipose tissue and BMI [31].

Nevertheless, other research has demonstrated that weight loss in individuals with obesity characterized by predominantly white and brown adipose tissue leads to a reduction in ACE2 mRNA expression in subcutaneous adipose tissue. It remains unclear whether these changes in ACE2 mRNA levels correspond to alterations in ACE2 protein expression or activity. Determination of ACE2 expression and/or activity in adipose tissue through cohort studies could help clarify which tissue(s) contribute to the observed reduction in systemic ACE2 levels, as well as the levels of Ang (1-7) and its substrate, des-Arg9-bradykinin.

Thus, circulating RAS and KKS peptides have been shown to be potential biomarkers of obesity that could aid in identifying adolescents who are at greater risk of developing obesity-related complications [32]. Evaluating the expression and activity of the main enzymes responsible for generating or inactivating RAS and KKS peptides, especially ACE and ACE2, are relevant points of interaction between the two systems. Additionally, elucidating the roles of RAS and KKS in the pathophysiology of obesity will contribute to the development of new therapeutic and prognostic strategies [31].

## 5. Physio-Pathological Links Between Obesity, Gut Microbiota and Intestinal Function

Previous studies have established a link between adiposity and the composition of the metabolome and gut microbiome. It has also been found that early exposures, such as mode of birth and breastfeeding, can influence gut microbiota composition in infants and increase the risk of childhood obesity [33]. Microbiota can influence how host metabolism functions by directly impacting energy and nutrient availability as well as modulating signaling pathways. Identifying potential biomarkers in the pediatric population through metabolomics (the scientific investigation of chemical processes that encompass metabolites, along with small molecule substrates, intermediates, and byproducts of cellular metabolism) and 16S rDNA profiling could present an opportunity to recognize these conditions and discover possible prevention and treatment strategies [34].

The human gut microbiome is primarily composed of two dominant bacterial phyla, Firmicutes and Bacteroidetes, which together represent over 90% of the total microbial population. Additionally, other less abundant phyla, such as Proteobacteria, Verrucomicrobia, and Actinobacteria, are also present. Although the composition of the gut microbiota is relatively stable during acute conditions, it can be affected by stressors associated with modern lifestyles, such as the consumption of food additives, chlorinated water, heavy metals, pesticides, antibiotics and organic pollutants [35,36]. These factors can lead to chronic changes in the composition of the gut microbiota, favoring the growth of more virulent microorganisms, with negative effects on host health. Intestinal dysbiosis is linked to a range of pathological conditions impacting the immune system (including type 1 diabetes, allergies, inflammatory bowel disease, and multiple sclerosis), the gastrointestinal tract (such as irritable bowel syndrome and diarrhea), the central nervous system (including autism, Parkinson’s, and Alzheimer’s), and the host’s energy homeostasis (such as atherosclerosis, type 2 diabetes, and obesity) [37].

Research has suggested that obese individuals present lower gut microbial diversity compared to normal-weight people and that weight loss leads to an increase in this diversity. Nevertheless, numerous studies have been contradictory and criticized for having small sample sizes and lacking sufficient statistical power to identify modest correlations [33].

Alterations in the relative abundance of the dominant phyla Firmicutes and Bacteroidetes were initially observed in obese animals, where an increase in Firmicutes was found at the expense of Bacteroidetes. Upon subjecting these animals to a calorie-restricted diet for one year, an increase in Bacteroidetes was noted, alongside a normalization of the Firmicutes/Bacteroidetes ratio (F/B ratio), which corresponded with weight loss. These findings led to the conclusion that Firmicutes are more efficient than Bacteroidetes in extracting energy from food, thereby facilitating more efficient calorie absorption and promoting weight gain. Such data suggest that changes in the composition and diversity of the gut microbiota are closely linked to alterations in its metabolic profile, which, in turn, can influence the health of the host. As a result, the Firmicutes/Bacteroidetes ratio has been increasingly proposed over the past decade as a potential biomarker of obesity [37].

The gut microbiome in healthy individuals has been shown to comprise a high Bacteroidetes/Firmicutes ratio, whereas in obese individuals, this ratio is inverted (with a higher abundance of Firmicutes). Furthermore, obese individuals have been found to exhibit elevated levels of Lactobacillus species, alongside a relatively reduced abundance of *Bacteroides vulgatus* [38].

Nonetheless, in contrast to these results, several studies have observed no change in this parameter or even reported a decrease in the F/B ratio in obese animals and humans [39].

In 2020, Magne et al. conducted a study to evaluate the validity of the F/B ratio as a potential marker, addressing the numerous conflicting findings in the literature. They concluded that the relative abundance of these phyla varies significantly among individuals within the same population, likely influenced by various lifestyle factors, including physical activity, diet, antibiotic consumption, food additives, and others, all of which contribute to the composition of the gut microbiota [40]. Although the gut microbiota may contribute to obesity development, the evidence supporting a clear association between obesity and changes in the F/B ratio remains inconclusive [37].

Short-chain fatty acids (SCFAs) are metabolic products generated by anaerobic fermentation of indigestible carbohydrates, including dietary fiber, resistant starch, and other polysaccharides, by gut microbiota within the small intestine and colon. The majority of acetate and propionate are synthesized by Bacteroidetes, while Firmicutes primarily generate butyrate. Short-chain fatty acids exert physiological effects on the host by interacting with free fatty acid receptors GPR41 and GPR43. Upon activation, these receptors promote the secretion of PYY (pancreatic peptide YY), a hormone associated with satiety, and enhance gastrointestinal motility. Activation of GPR41 further stimulates leptin production in adipocytes and accelerates liver fat synthesis. In addition, butyrate and propionate are involved in appetite suppression by stimulating the release of the gut hormone GLP-1 (glucagon-like peptide) [41].

A study involving 52 Czech children and adolescents, aged 7 to 16 years, analyzed stool samples to explore potential metabolomics and gut microbiota biomarkers linked to childhood overweight and obesity. The results indicated higher levels of fecal butyrate in the obese patient group compared to the normal-weight group, reinforcing earlier findings that show increased concentrations of SCFAs in overweight/obese children compared to their normal-weight peers [42].

Another study of 170 two-year-old children led by Nandy et al. also showed that there is a positive correlation between stool butyrate concentration and a child’s weight gain, and this should be further investigated as a factor affecting childhood obesity [40].

Multiple mechanisms could account for the observed increase in fecal butyrate, including: (1) amplified abundance of carbohydrate-fermenting gut bacteria in obese individuals, which subsequently leads to increased SCFA production, an increase which may provide the host with surplus energy that can be stored as lipids or glucose; and (2) decreased absorption caused by accelerated intestinal transit or low-grade inflammation.

It is considered that this metabolite may play an important role in maintaining intestinal balance and may be involved in the communication between the gut and the brain via the gut–brain axis. In 2016, in a study by Goffredo et al. involving 84 young people (ranging from normal weight to morbidly obese), it was found that the three primary SCFAs exhibited a positive correlation with body and visceral fat, and among these, butyrate was the only SCFA significantly linked to liver fat [43].

While the elevated concentration of SCFAs in overweight and obese children, as identified in several studies, may imply their role as biomarkers of obesity, this interpretation remains controversial. SCFAs are known to confer various benefits, including improved blood lipid profiles, enhanced glucose homeostasis, and potential body weight reduction. A comparable concept can be drawn from nutrient overload: a certain level of nutrients might be beneficial, but excessive amounts could have detrimental effects [42].

Houtman et al. conducted a 14-year longitudinal study to investigate the validity of the Firmicutes/Bacteroidetes (F/B) ratio and short-chain fatty acid precursors in relation to body mass index (BMI) in a growth and development context. Study results showed limited evidence supporting a relationship between the F/B ratio and BMI, as well as between the sum of known SCFA precursors and BMI during the first 12 years of life. Particularly during childhood, the F/B ratio may be unreliable because Bacteroides are not present in sufficiently high proportions. The absence of Bacteroidetes in the childhood sample could potentially be attributed to geographical factors. Nevertheless, no association was identified between the F/B ratio and indexed BMI (body mass index) at ages between six and ten, proving that results are not solely attributed to the absence of Bacteroidetes in childhood. Preliminary investigations indicated that Firmicutes and Bacteroidetes were linked to changes in indexed BMI over the first 12 years of life, and the SCFA-producing genera Alistipes (propionate) and Subdoligranulum (butyrate) were inversely associated with future indexed BMI during childhood. Therefore, it is not currently advised to use the Firmicutes-to-Bacteroides ratio or short-chain fatty acid precursors as a broad-spectrum predictor biomarker for childhood BMI, especially in infants [44].

Fecal calprotectin (FC) is a macromolecule released by neutrophils and macrophages in the intestinal mucosa utilized to detect intestinal inflammation. FC has a sensitivity rate of 83% and a specificity of 84% to distinguish organic from non-organic diseases [45]. In a study by Spagnuolo et al. on a group of 34 severely obese children (BMI ≥ 95%), about one-third of the children were found to have elevated fecal calprotectin levels, exceeding 100 μg/g. Also, in two instances of very severe obesity, levels exceeded 200 μg/g, approaching levels measured in children with irritable bowel syndrome. These findings suggest the existence of chronic inflammation of the intestine in severely obese children, even in the absence of specific bowel manifestations and bowel disease. Additionally, the levels of FC and CRP lie between those of normal intestinal mucosa and those of active inflammation (such as those seen in inflammatory bowel disease) [46]. It is widely recognized that obesity is linked to asymptomatic inflammation even among children and adolescents. Although most studies on obesity and inflammation focus on adults and include the assessment of fecal calprotectin levels, detailed research involving children of different age groups is needed due to rapid development and variation in lifestyles by age group. Results from a study by Jun Hwan Kim revealed that fecal calprotectin levels were reduced in relation to obesity during middle childhood but were not considerably reduced in adolescents. The previously mentioned findings suggest that the impact of intestinal inflammation on inflammatory bowel disease (IBD) increases with maturity in children, particularly in obese children with IBD. The study also found that FC concentrations were reduced in obese children with irritable bowel syndrome, and the grade of obesity affected gut response to inflammation in adults differently than in pediatric patients. Therefore, it is hypothesized that the pathophysiological mechanisms underlying IBS in children might be different from those in adults, especially in terms of the interaction between obesity and intestinal inflammation [47].

In a study conducted by Park and Kim on a sample of 74 subjects, including children and adults, the correlation between intestinal immune response and obesity/overweight was assessed using fecal calprotectin levels. According to the study results, FC revealed a meaningful difference between overweight and obese adults and children but showed no significant difference between normal-weight adults and children. Additionally, FC levels were decreased in overweight and obese children compared to normal-weight children but were higher in overweight and obese adults than in normal-weight adults. Such findings suggest that the pathophysiological mechanisms involved in obesity in children and adults are not the same. FC is secreted by stimulated neutrophils, with its values in healthy children tending to decrease with age and reaching adult levels by the age of four. However, the exact causes of the variation in FC levels with age are not yet fully understood and require further investigation [48].

## 6. Discussion

In this article, we aim to highlight the importance of markers and mechanisms that can enhance specialists’ understanding of obesity assessment and its systemic complications. According to various statistics from the World Obesity Federation, it is estimated that by 2025, a little over 200 million children and adolescents will be diagnosed with childhood obesity, with an upward trend that will culminate in 254 million by 2030. In Europe, including Romania, the situation remains just as worrying, with the prevalence of obesity rising from 10% in 2010 to 18.5% in 2018 [23,49].

Biomarkers are biological indicators associated with certain pathologies, the most incriminated in childhood obesity being biochemical, salivary, and fecal biomarkers. Biochemical biomarkers include CRP, ferritin, leukocytes, and red cell width distribution. Victorio Higgins et al. emphasized in 2020 the direct link between increased CRP levels in relation to excess adipose tissue, and Antonella S. Fittipaldi furthered the study by directly implicating CRP in the progression of cardiac and vascular diseases, especially myocardial infarction [10].

Adipose tissue plays a role in the onset and persistence of systemic inflammation, with neutrophils, macrophages, mast cells, and white blood cells involved in shaping the immune response and also in the advancement of metabolic syndrome. The most recently discovered indicator for the diagnosis of inapparent inflammation is the neutrophil/lymphocyte ratio, which is correlated with the development of inflammatory and cardiovascular diseases and cancer.

Agostini Sobrinho et al. highlight in a study conducted in 2022 the role of leptin and adiponectin in the development of childhood obesity, being connected to variations in immune function and glucose metabolism [13]. Idalia Cura-Esquivel (2023) states that both obesity and metabolic syndrome are characterized by reduced serum adiponectin levels alongside elevated circulating leptin, leaving room for future studies [2]. Adipokines are released by adipose tissue and take part, through autocrine and paracrine mechanisms, in the management of energy consumption, insulin responsiveness, glucose and lipid metabolism, and not least endothelial activity and immune response.

Selvaraju V. et al. in 2019 highlighted the role of resistin, an adipokine produced by macrophages in the pathogenesis of inflammation, which is found predominantly in patients diagnosed with childhood obesity [19]. Hypertension and cardiovascular disease are closely linked to obesity, as the RAS (renin–angiotensin system) has two pathways that produce opposing physiological effects and has multiple locations, such as cardiac, nervous, pancreatic, and adipose tissues. RAS and KKS take part in the pathophysiology of several diseases such as diabetes, Alzheimer’s disease, and metabolic syndrome and are also modeled in childhood obesity.

Salivary markers are innovative options in the diagnosis of childhood obesity, and in recent years they are increasingly being used in its early diagnosis. They have many advantages, including sampling and analytical advantages, but also some disadvantages in relation to pediatric patient status.

Hend Alqaderi et al. in 2022 conducted a study highlighting the role of pro-inflammatory molecules IL-6, IL-8, IL-10, leptin, CRP, and adiponectin determined at the salivary level in the evaluation of pediatric obesity [24]. Increases in CRP and insulin were correlated with percentage increases in BMI, with insulin being a predictor of hyperglycemic status [24]. Complementing the above study, Corina Pursean et al. identified up to a 4.5-fold increase in salivary IL-6 in the overweight group.

Other markers possibly involved in the diagnosis of childhood obesity are adiponectin and fetuin A, the latter having increased values in relation to BMI, while adiponectin is inversely associated with body mass index, according to a 2022 study conducted by Hend Alqaderi et al. [24]. Last but not least, the identification and determination of salivary uric acid in a group of pediatric patients diagnosed with childhood obesity were correlated with reduced insulin sensitivity status and the occurrence of cardiometabolic syndrome.

The determination of salivary uric acid may represent an innovative pre-diagnostic method, but research is still in its early stages. Another factor in the early prognosis of childhood obesity is the gut microbiota. Firmicutes and Bacteroidetes, the two dominant bacterial phyla, make up over 90% of the total. An association between adipose tissue and gut microbiome composition has been demonstrated. There are many factors, such as mode of birth or breastfeeding, that alter the structure of the gut microbiota and thus increase the risk of developing obesity in the future.

Gut microbial diversity is lower in obese people, with weight loss having a beneficial effect on increasing its diversity. Magne F. et al. emphasized in a 2020 study the involvement of the two bacterial phyla, with obese patients showing higher amounts of Firmicutes compared to Bacteroidetes. Subjects were put on a hypocaloric diet, subsequently showing an increase in Bacteroidetes and normalization of the Firmicutes/Bacteroidetes ratio [37]. Thus, changes in Bacteroidetes composition were linked to alterations in the metabolic profile, with the Firmicutes-to-Bacteroidetes proportion being considered a possible pre-diagnostic marker for obesity.

## 7. Limitations and Strengths

This review provides a comprehensive and interdisciplinary assessment of obesity biomarkers that integrates biochemical markers, salivary and fecal markers with systemic inflammation, metabolic syndrome, and gut microbiota interactions. A key strength is its focus on non-invasive biomarkers, offering potential for early management of obesity. It also covers RAS and KKS in obesity complications, adding depth to the topic. However, the review has limitations, including a lack of critical discussion on conflicting evidence, particularly on the Firmicutes/Bacteroidetes ratio. It also does not fully explore factors like diet, genetics, and socioeconomic status that may influence biomarker levels. Additionally, most cited studies are observational, limiting conclusions on causality. Future research should focus on longitudinal and interventional studies to strengthen biomarker validation in pediatric obesity.

## 8. Conclusions

In conclusion, the evaluation of childhood obesity is, without a doubt, a critical step in preventing its rising prevalence and associated health risks. Biomarkers, such as biochemical, salivary, and fecal markers, offer promising methods for quick detection and prognosis. Inflammatory markers known as CRP, leptin, adiponectin, and the neutrophil/lymphocyte ratio have shown significant correlations with disorders related to obesity. Additionally, the composition of the gut microbiota, especially the Firmicutes/Bacteroidetes ratio, presents a good potential for pre-diagnostic evaluation. The growing body of evidence highlights the important role of these biomarkers in monitoring childhood obesity, providing valuable information about inflammation, metabolic health, and cardiovascular risks. Furthermore, the gut microbiota, with its Firmicutes/Bacteroidetes ratio, stands out as a promising area for future studies with the goal of improving diagnosis and therapy.

## Figures and Tables

**Table 1:** Biomarkers currently linked to obesity.

Biomarker	Type	Function/Association
C-reactive protein (CRP)	Serum	Inflammation and cardiovascular risk
Ferritin	Serum	Inflammatory marker
White blood cell count	Serum	Inflammatory marker
Red cell distribution width	Serum	Inflammatory marker
Tumor necrosis factor-alpha (TNF-α)	Serum	Inflammation and insulin resistance
Interleukin-6 (IL-6)	Serum	Inflammation and insulin resistance
Neutrophil-to-lymphocyte ratio (NLR)	Serum	Subclinical inflammation marker
Adiponectin	Serum	Regulates insulin sensitivity, inversely related to obesity
Leptin	Serum	Regulates appetite and metabolism, increased in obesity
Ghrelin	Serum	Appetite hormone, increases hunger
Fetuin-A	Serum	Involved in appetite control and insulin regulation
Insulin	Serum	Hyperinsulinemia marker
Resistin	Serum	Inflammation marker
Salivary IL-6	Salivary	Pro-inflammatory cytokine
Salivary IL-8	Salivary	Pro-inflammatory cytokine
Salivary IL-10	Salivary	Anti-inflammatory cytokine
Salivary leptin	Salivary	Appetite regulation, increased in obesity
Salivary CRP	Salivary	Inflammatory marker
Salivary insulin	Salivary	Predictor of hyperglycemic status
Salivary VEGF	Salivary	Vascular endothelial growth factor, metabolic association
Salivary MCP-1	Salivary	Monocyte chemoattractant protein-1, immune regulation
Salivary adiponectin	Salivary	Anti-inflammatory, inversely related to obesity
Salivary uric acid	Salivary	Correlated with insulin resistance and metabolic syndrome
Angiotensin I (Ang I)	Renin–Angiotensin System	Regulates blood pressure, associated with obesity
Angiotensin II (Ang II)	Renin–Angiotensin System	Regulates blood pressure, associated with obesity
Angiotensinogen	Renin–Angiotensin System	Precursor protein in RAAS
Ang1-7	Renin–Angiotensin System	Anti-inflammatory RAS component
Bradykinin	Renin–Angiotensin System	Vasodilator peptide
Des-Arg9-bradykinin	Renin–Angiotensin System	Inflammatory kinin
Firmicutes/Bacteroidetes Ratio	Gut Microbiota	Gut microbiome ratio linked to obesity
Short-chain fatty acids (SCFAs)	Gut Microbiota	Metabolites involved in obesity and energy regulation
Fecal calprotectin (FC)	Fecal	Intestinal inflammation marker, associated with obesity
